# Synthesis and Characterization of Carvedilol-Etched Halloysite Nanotubes Composites with Enhanced Drug Solubility and Dissolution Rate

**DOI:** 10.3390/molecules28083405

**Published:** 2023-04-12

**Authors:** Lauretta Maggi, Claudia Urru, Valeria Friuli, Chiara Ferrara, Debora Maria Conti, Giovanna Bruni, Doretta Capsoni

**Affiliations:** 1 Department of Drug Sciences, University of Pavia, Via Taramelli 12, 27100 Pavia, Italy; lauretta.maggi@unipv.it (L.M.); valeria.friuli@unipv.it (V.F.); 2 Department of Chemistry, Physical Chemistry Section & C.S.G.I. (Consorzio Interuniversitario per lo Sviluppo dei Sistemi a Grande Interfase), University of Pavia, 27100 Pavia, Italy; claudia.urru01@universitadipavia.it (C.U.); deboramaria.conti01@universitadipavia.it (D.M.C.); giovanna.bruni@unipv.it (G.B.); 3 Department of Materials Science, University of Milano-Bicocca, Via Cozzi 55, 20125 Milano, Italy; chiara.ferrara@unimib.it

**Keywords:** halloysite nanotubes, carvedilol, dissolution tests, drug–nanoclay composites, solid-state NMR

## Abstract

Carvedilol is a poorly water-soluble drug employed to treat chronic heart failure. In this study, we synthesize new carvedilol-etched halloysite nanotubes (HNTs) composites to enhance solubility and dissolution rate. The simple and feasible impregnation method is used for carvedilol loading (30–37% weight). Both the etched HNTs (acidic HCl and H_2_SO_4_ and alkaline NaOH treatments) and the carvedilol-loaded samples are characterized by various techniques (XRPD, FT-IR, solid-state NMR, SEM, TEM, DSC, and specific surface area). The etching and loading processes do not induce structural changes. The drug and carrier particles are in intimate contact and their morphology is preserved, as demonstrated by TEM images. The ^27^Al and ^13^C solid-state NMR and FT-IR findings show that carvedilol interactions involve the external siloxane surface, especially the aliphatic carbons, the functional groups, and, by inductive effect, the adjacent aromatic carbons. All the carvedilol–halloysite composites display enhanced dissolution rate, wettability, and solubility, as compared to carvedilol. The best performances are obtained for the carvedilol–halloysite system based on HNTs etched with HCl 8M, which exhibits the highest value of specific surface area (91 m^2^ g^−1^). The composites make the drug dissolution independent of the environmental conditions of the gastrointestinal tract and its absorption less variable, more predictable, and independent from the pH of the medium.

## 1. Introduction

It is established that the poor water solubility of drugs represents a critical issue for the pharmaceutical industry [1], as solubility is crucial to achieving proper bioavailability for orally administered drugs. It is assessed that 40% of the drugs currently marketed and 70–90% of those under development display low solubility in water [2]. A huge variety of techniques has been applied to improve drug solubility and dissolution rates [3]. They are mainly based on reducing particle size, increasing the specific surface area, and stabilizing different forms, such as polymorphs [4,5], hydrates, solvates [6,7,8], salts [9,10,11], and co-crystals [1,12,13,14,15]. In addition, complexation [16] and encapsulation methods [17,18,19] have proven to be viable and effective strategies to enhance the solubility and dissolution rate of many drugs.

In recent years, the increased interest in nanoscience and nanotechnology stimulated researchers to develop new carriers with peculiar properties as suitable vehicles for drugs and efficient platforms for poorly soluble active principles. Improved solubility, dissolution rate, and drug release systems were obtained by employing carriers such as liposomes, nanocapsules, carbon nanotubes, and naturally occurring nanostructured materials, such as nanoclays [20].

Halloysite is a natural aluminosilicate clay belonging to the kaolin group with the chemical formula Al_2_Si_2_O_5_(OH)_4_·nH_2_O. Two crystalline forms are reported in the literature: the di-hydrated phase (halloysite-10 Å) easily releases the water molecules to form the anhydrous one (halloysite-7 Å), commonly observed at room temperature [21,22]. Halloysite displays a bi-layered structure, in which the SiO_4_ tetrahedra sheet connects via oxygen to the AlO_6_ octahedra one. Three possible morphologies are reported for halloysite: nanotubular, platy sheets (typical of kaolinite, too), and spheroidal; among them, the former is commonly observed as the mismatch of the oxygen-sharing tetrahedral and octahedral sheets are responsible for the layer wrapping and tubular arrangement [23,24]. With typical lengths of 0.4–1 μm, external diameter of 20–200 nm, and internal diameter of 10–70 nm [25], the halloysite nanotubes (HNTs) display a high aspect length/diameter ratio and surface area of about 50 m^2^ g^−1^ [26]. Notably, the siloxane and silanol groups on the external surface and the aluminum groups on the internal one make the two surfaces negatively and positively charged, respectively. This ensures easy interactions between halloysite and various synthetic and biological molecules [27,28]. The impressive properties of halloysite can be further improved by functionalization. Different approaches were applied, such as acid and alkali activation [29,30], intercalation [31,32], thermo-chemical treatment, and chemical modification [33,34].

Furthermore, in vitro cytotoxicity [35] and ecotoxicity investigations [36] demonstrated expanded biocompatibility and low toxicity of halloysite, thus making this nanoclay an ideal platform to vehiculate drugs.

**Scheme 1:**
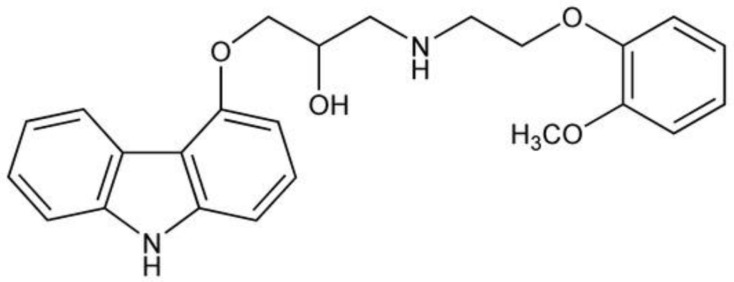
Molecular structure of carvedilol.

Carvedilol ((2RS)-1-(9H-Carbazol-4-yloxy)-3-[[2-(2-methoxyphenoxy)ethyl] amino] propan-2-ol) is an active principle with the chemical formula C_24_H_26_N_2_O_4_ and molecular structure shown in Scheme 1.

The drug is used to alleviate tardive movement disorders, psychosis, mania, and depression [37,38]. Still, it is mainly applied as a β—blocker and vasodilator to treat cardiovascular disorders, such as angina pectoris, cardiac arrhythmias, and myocardial infarction. It is proven to increase life expectancy in patients with chronic heart failure [39,40]. 

Carvedilol is highly soluble in dimethyl sulfoxide, soluble in dichloromethane and methanol, moderately soluble in ethanol and isopropanol, and poorly soluble in di-ethylic ester. As concerns aqueous media, it displays good permeability through the gastrointestinal membrane, but it is practically insoluble in water and simulated intestinal fluid (without pancreatin, pH 7.5). At the same time, it is slightly soluble in simulated gastric fluid at pH 1.0. For these reasons, it can be classified as a class II drug in the Biopharmaceutics Classification System (BCS). It is a weak base (pKa = 7.8) in the 1–8 pH range; its poor solubility increases from 0.01 to 1 mg mL^−1^ by decreasing pH [41,42]. Various approaches were investigated to enhance carvedilol’s solubility and dissolution rate, such as cyclodextrin complexation, chitosan–carvedilol systems, solid dispersion, anti-solvent precipitation, and co-crystallization [39,40,43,44,45,46].

This paper reports the synthesis, characterization, and dissolution behavior of carvedilol halloysite and carvedilol-etched halloysite nanotubes composites. To our knowledge, these systems are not yet investigated and are obtained by an impregnation method, a simple and viable synthetic approach. Both acidic (HCl and H_2_SO_4_ solutions in different concentrations) and alkaline media were employed to activate commercial halloysite. Carvedilol was loaded on both commercial and etched halloysite, and all the samples were characterized by XRPD and FT-IR and tested for dissolution behavior. The samples representative for each system were further investigated by SEM, TEM, DSC, specific surface area, ^27^Al and ^13^C solid-state NMR, solubility, and wettability (contact angle) to evaluate morphology and elucidate the drug–halloysite interactions and properties.

## 2. Results and Discussion

Commercial HNTs were activated by acidic (HCl or H_2_SO_4_) or alkaline (NaOH) etching. Different acidic concentrations were used. All the etched samples were characterized and compared to the commercial HNTs. The impregnation method was employed for loading carvedilol on both commercial and etched halloysite. In addition, some carvedilol–halloysite physical mixtures were prepared to compare the dissolution behavior (PM samples). Details on the synthesis procedure and characterization techniques are reported in Section 3. A list of the synthesized samples and the alphanumeric codes used hereafter is reported in Table 1. 

As can be seen from Table 1, several etched and carvedilol-loaded samples were prepared. For sake of simplicity, we decided to report the characterization results of all the samples in Supplementary Materials. In this chapter, we show and discuss the results of the samples giving the best dissolution behavior for each etching system and suitable to examine the influence of the etching on the dissolution performances of carvedilol. The chosen systems are H_HCl_8M, H_H_2_SO_4__0.5M, H_NaOH_0.5M, and their composites with carvedilol. The carvedilol (C), commercial HNTs (H), and carvedilol–halloysite physical mixtures are also discussed for comparison. 

### 2.1. Sample Characterization

#### 2.1.1. XRPD

**Figure 1:**
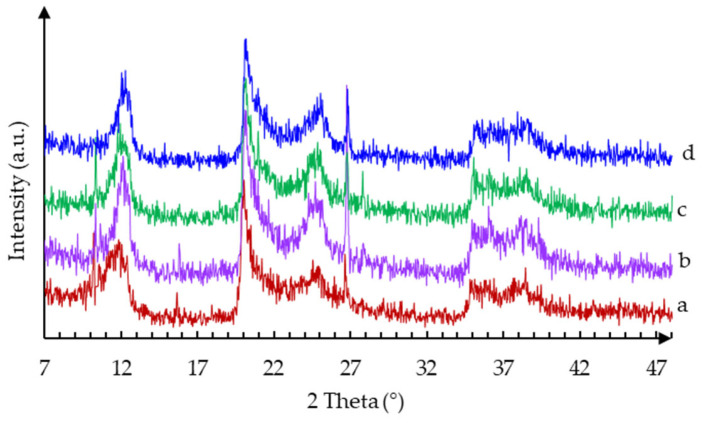
XRPD pattern of H (a), H_HCl_8M (b), H_H_2_SO_4__0.5M (c), and H_NaOH_0.5M (d).

In Figure 1, the X-ray powder diffraction pattern of the commercial halloysite is shown and compared to the etched ones. The diffraction peaks match those reported in the JCPDS Database for halloysite (PDF# 029-1487). According to Bragg’s law, the basal plane at 12° (*d* spacing: 7.35 Å) corresponds to the (0 0 1) reflection and it is peculiar to the anhydrous halloysite phase; the peak at about 24° is assigned to the (0 0 2) reflection (*d* = 3.63 Å). Further confirmation of the absence of hydration water comes from the lack of the peak at 8.8°, indicative of the 10 Å hydrated halloysite [47]. The broad peak at about 20° is attributed to the (1 0 0) reflection and confirms the nanotubular structure of the halloysite employed [48,49]. The peaks at about 35 and 37.9° are assigned to the (1 1 0) and (0 0 3) reflections, respectively. Sharp peaks at about 10.1°, 23.8°, and 26.6° suggest the presence of kaolinite 1A, rutile, and quartz, respectively, as a small quantity of impurities typically detected in halloysite minerals. Activated samples do not show changes in the crystalline and nanotubular structure of the halloysite, regardless of the etching agent employed (Figure 1) and its concentration (Figure S1). Indeed, the XRPD patterns of all the investigated samples well compare to the literature ones [50,51].

**Figure 2:**
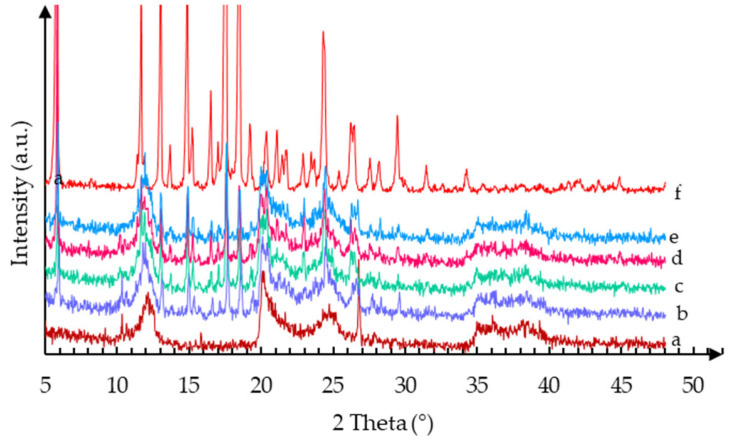
XRPD pattern of H (a), CH, (b) CH_HCl_8M (c), CH_H_2_SO_4__0.5M (d) CH_NaOH_0.5M (e), and C (f).

Figure 2 displays the patterns of carvedilol alone and loaded onto HNTs for the CH, CH_HCl_8M, CH_H_2_SO_4__0.5M, and CH_NaOH_0.5M samples. The commercial halloysite diffraction pattern is also shown for comparison. The carvedilol pattern shows narrow diffraction peaks, suggesting that the sample is highly crystalline. The peak positions agree with those reported in the literature for crystalline form II [41]. According to Prado et al. [52], carvedilol form II displays a monoclinic structure with P2_1_/c *S.G.* and lattice parameters *a* = 15.5414 Å, *b* = 15.2050 Å, *c* = 9.1174 Å, and *β* = 100.73° at 173 K. The most intense peaks at 5.8°, 11.6°, 13.0°, 14.8°, 16.5°, 18.4°, 19.2°, 24.3°, 26.5°, and 29.6° are assigned to the (1 0 0), (0 2 0/2 0 0), (1 2 0/2 1 0), (2 −1 −1), (2 2 0), (1 3 0/3 1 0), (3 −1 −1), (3 2 1/4 −1 −1), (3 −2 −2/1 4 1), and (5 0 0/4 −3 −1) reflections, respectively. Drug–clay patterns show the presence of both halloysite and carvedilol peaks, confirming the successful loading and demonstrating that neither decomposition nor crystalline structure modifications occurred. These findings are also supported by the XRPD patterns of the other carvedilol-loaded (Figure S2) and physical mixture samples (Figure S3). Moreover, the results agree with the NMR investigation (Section 2.1.3 and Figure S4 in the Supplementary Materials) carried out for the same samples at the ^27^Al frequencies, revealing that all the samples present an unaltered structure. Thus, the halloysite structure is maintained unmodified from the short- and long-range points of view.

#### 2.1.2. FT-IR Spectroscopy

**Figure 3:**
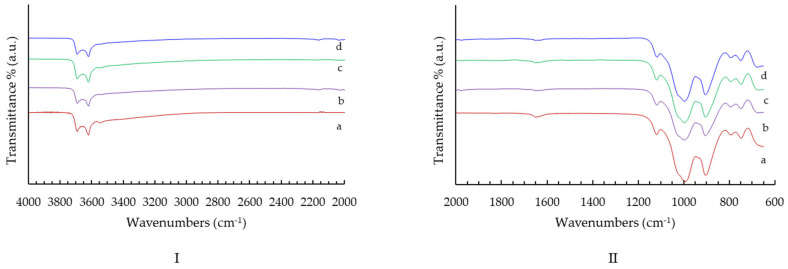
FT-IR spectra of H (a), H_HCl_8M (b), H_H_2_SO_4__0.5M (c), and H_NaOH_0.5M (d) in 4000–2000 cm^−1^ (**I**) and 2000–600 cm^−1^ (**II**) wavenumber ranges.

Figure 3 shows unmodified and etched halloysite spectra (H_HCl_8M, H_H_2_SO_4__0.5M, and H_NaOH_0.5M samples). Commercial halloysite spectrum (Figure 3, spectrum (a)) well compares to those reported in the literature [53,54]. The weak bands at about 3540 cm^−1^ and 1650 cm^−1^, mainly detected in the H spectrum, suggest the presence of traces of interlayer water molecules not detectable with the XRPD technique. As reported in the literature [48,55], the water molecules are weakly linked to the halloysite and can be easily removed from the structure near room temperature. The spectra on the treated samples (Figure 3, spectra (b)–(d)) well compare to the H one. FT-IR results further confirm that acid or alkaline treatment does not influence the halloysite’s absorption bands, and the halloysite structure is preserved, as suggested by the XRPD results.

**Figure 4:**
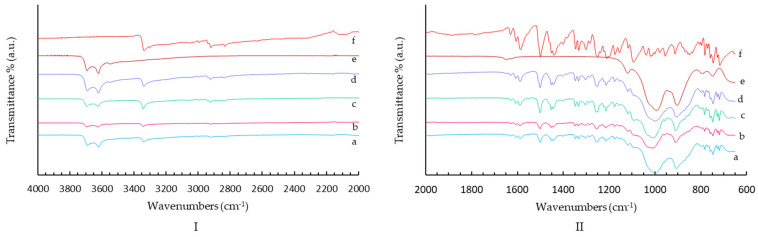
FT-IR spectra of CH_NaOH_0.5M (a), CH_H2SO4_0.5M (b), CH_HCl_8M (c), CH (d), H (e), and C (f) in 4000–2000 cm^−1^ (**I**) and 2000–600 cm^−1^ (**II**) wavenumber ranges.

The carvedilol spectrum (Figure 4, spectrum (f)) well compares to those reported in the literature. Notably, the band related to the alcoholic -OH stretching stands at 3335 cm^−1^, confirming that carvedilol is the crystalline form II [41]. 

Drug–clay systems spectra (Figure 4, spectra (a)–(d)) show the simultaneous presence of both halloysite (Figure 4, spectrum (e)) and carvedilol (Figure 4, spectrum (f)) signals, supporting the hypothesis of a successful loading. 

A list of the absorption bands and ascribed groups of the commercial halloysite sample, carvedilol, H_HCl_8M (taken as an example for the treated samples), and CH_HCl_8M (taken as an example for the loaded samples) is summarized in Table S1. For completeness, the FT-IR spectra of all the investigated samples are shown in Figures S5–S7. Further details on possible carvedilol–halloysite interactions will be discussed and compared to the ^13^C solid-state NMR findings. 

#### 2.1.3. Solid-State NMR

**Figure 5:**
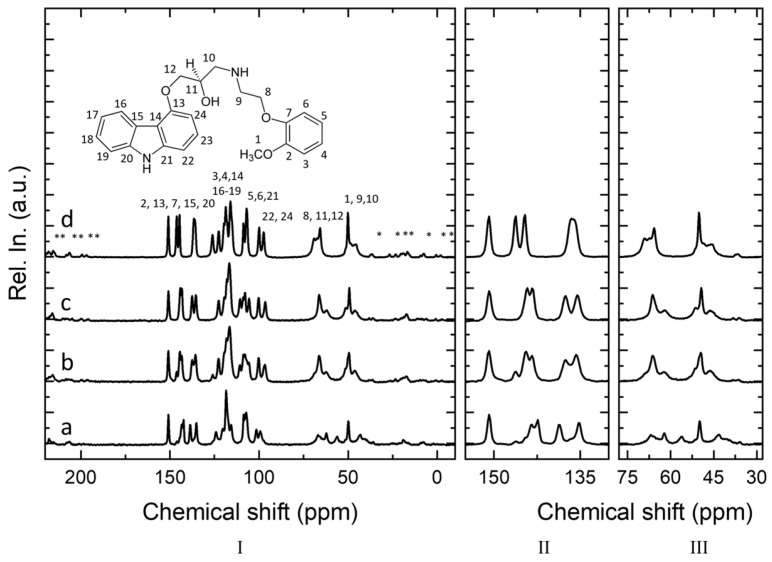
The ^13^C NMR spectra of CH_H_2_SO_4__0.5M (a), CH_HCl_8M (b), CH (c), and C (d) with attribution for the C sample in the 200-0 ppm range (**I**) and zoom in the 155-130 ppm range (**II**) and in the 75–30 ppm range (**III**) to highlight the regions presenting the more significant changes. The symbol * marks a spinning sideband.

The ^13^C solid-state NMR spectra for the commercial carvedilol sample, together with the selected loaded compositions, are reported in Figure 5. The spectrum obtained for the carvedilol (Figure 5, spectrum (d)) is compatible with its crystalline nature, as also evidenced by the XRPD analysis discussed above and is in good agreement with the previous report. Different reports of NMR studies have appeared in the literature [56,57,58]; the most reliable signal attribution is the one reported by Gadape et al. [56] with a detailed study on the quantification of carvedilol. The spectrum acquired for the CH commercial composition (Figure 5, spectrum (c)) is similar to the one registered for pure carvedilol. Still, it reveals some changes (i.e., signals related to C7, C15, C20 and C8, C11 and C12 in Figure 5II,III), indicating that the structure is still highly ordered but with different spatial conformation with respect to the pure carvedilol form. Similarly, the spectra obtained for the CH_H_2_SO_4__0.5M and CH_HCl_8M samples are similar to the pristine C material, indicating that no chemical modification and/or decomposition are involved in the loading procedure, thus confirming the suitability of the developed method for the sample preparation. At the same time, some differences are detected, in particular, peaks broadening, peaks shifting, and peaks doubling (see Figure 5II,III). These effects are associated with changes in the mobility of the involved moieties (enhanced broadening), changes in the magnetic shielding at local scale (peaks shift), and diversification of the local environment (peaks doubling). While the portion of the spectrum associated with aromatic moieties (130–100 ppm) is not greatly affected moving from pristine carvedilol to the loaded samples, the peaks related to the aliphatic portion of the molecules (75–30 ppm) and functional groups (160–130 ppm) are characterized by the major changes. Carbon C1, C9, C10, and C11 are probably involved in interaction with the halloysite component and/or with other carvedilol moieties, as inferred from the shifts in the associated signals. This is also reflected in the resonances associated with the C2, C13, and C7 positions as an inductive effect. Shifts and peaks splitting can also be observed in comparison with previous reports on carvedilol in various forms and preparations with different excipients [56,57,58,59] due to the changes in local environments and configuration of the molecular moieties and modifications in the local magnetic fields. Similar effects have been evidenced in several systems by our groups and rationalized in a similar way [60,61,62].

Nevertheless, a deeper analysis of the NMR spectra and rationalization of the changes is not possible only on the basis of the present data, and simulations would be required. However, the reported results suggest that possible changes in the IR spectra can be detected. We investigated in detail the FT-IR bands involving the discussed carbons for carvedilol, physical mixture, and carvedilol–halloysite hybrids. The band positions are reported in Table 2. They are very close for carvedilol and physical mixture samples, as expected. In fact, no halloysite–carvedilol interaction occurs in the physical mixture. In the case of loaded samples, the bands slightly shift, suggesting a possible interaction between active principle and halloysite. Finally, ^27^Al spectra have been acquired for the halloysite pristine compound and the two selected loaded samples (Figure S4) to verify that no modifications of the halloysite structure are involved in the loading procedure. The obtained spectra confirm that the halloysite structure is preserved. Indeed, the signal of the halloysite is a single resonance at −2 ppm, typical of AlO_6_ moieties, which is in good agreement with a previous report on halloysite and oxides samples [63,64,65]. The signal form of the loaded samples is essentially unaltered, suggesting that the intercalation of carvedilol moieties does not involve severe modification of the halloysite backbone. This is expected as the AlO_6_ units constitute the inner layer, and thus do not experience a direct interaction with the intercalated species.

In conclusion, the NMR data confirm the integrity of the carvedilol molecules and that their conformation is different in the three considered samples, which is in agreement with the XRPD and FT-IR analysis.

#### 2.1.4. SEM

**Figure 6:**
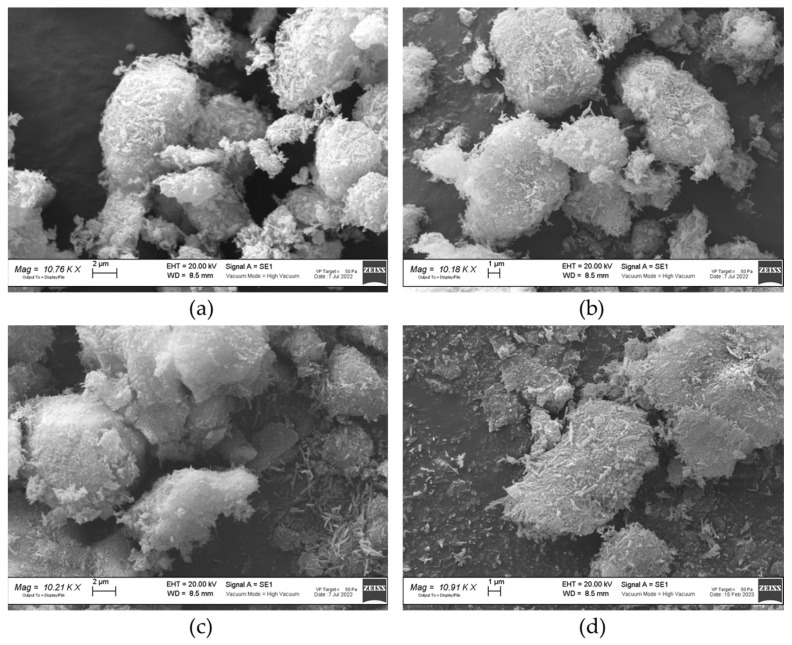
SEM images at 10 kX magnification of H (**a**), H_H_2_SO_4__0.5M (**b**), H_HCl_8M (**c**), and H_NaOH_0.5M (**d**).

SEM images of commercial HNTs are reported in Figure 6a. The sample is characterized by micrometric aggregates (2–20 µm) of needle-like particles. Etching treatments negligibly modify the nanotubular structure, as displayed in Figure 6b–d. 

**Figure 7:**
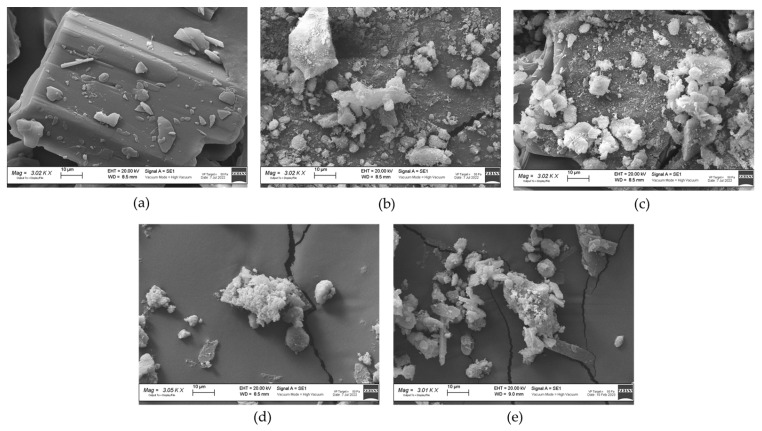
SEM images at 3 kX magnification of C (**a**), CH (**b**), CH_HCl_8M (**c**), CH_H_2_SO_4__0.5M (**d**), and CH_NaOH_0.5M (**e**).

In Figure 7, the SEM images of carvedilol and carvedilol–halloysite samples are shown. Carvedilol consists of micrometric squared particles (up to 100 µm in size) with a very smooth surface (Figure 7a). In drug–clay systems, HNTs seem to cover carvedilol particles. This is possibly due to the establishment of weak surface interactions (Figure 7b–e), as suggested by IR and NMR results. SEM analysis is also performed for physical mixture samples, as shown in Figure S8; in these cases, HNTs do not cover the carvedilol surface but form isolated clusters. For a better understanding of morphologies and active principle–halloysite distribution in the samples, TEM analysis is performed.

#### 2.1.5. TEM

**Figure 8:**
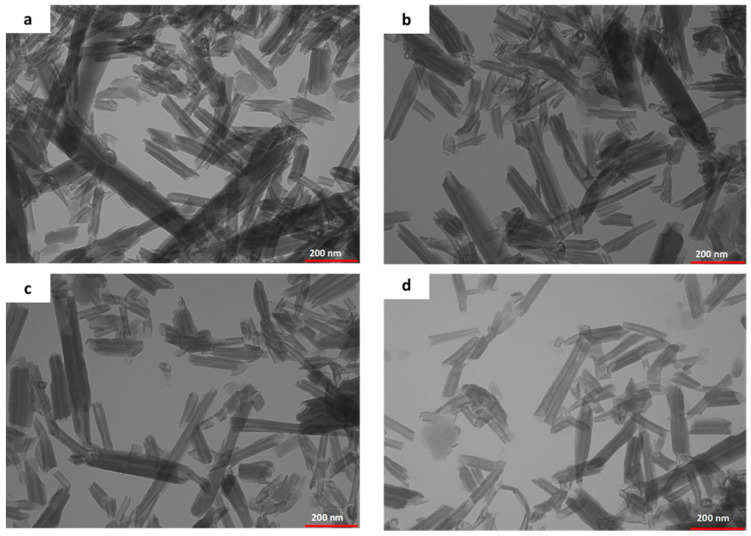
TEM images at 100 kX magnification of H (**a**), H_HCl_8M (**b**), H_H_2_SO_4__0.5M (**c**), and H_NaOH_0.5M (**d**).

TEM images of commercial HNTs confirm their nanotubular structure. Nanotubes are characterized by an external diameter ranging from 50 to 80 nm and a constant lumen sized 15 nm. The etching treatments do not alter the nanotubular structure in all treated samples (Figure 8). The alkaline etching tends to disaggregate clusters. 

**Figure 9:**
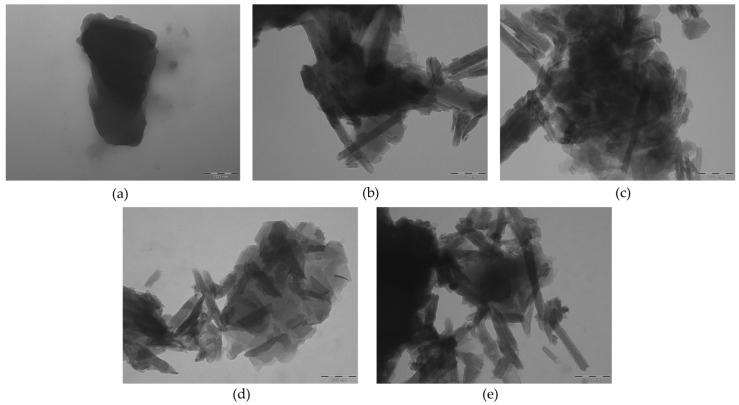
TEM images at 100 kX magnification of C (**a**), CH (**b**), CH_HCl_8M (**c**), CH_H_2_SO_4__0.5M (**d**), and CH_NaOH_0.5M (**e**).

In Figure 9, the TEM images of the carvedilol and drug–clay samples are shown. Carvedilol (Figure 9a) displays squared particles whose morphology is consistent with that observed in SEM micrographs (Figure 7a). Interestingly, in all drug–clay samples (Figure 9b–e), agglomerates of strongly interconnected carvedilol particles and halloysite nanotubes are detected. This evidence confirms the intimate contact between the drug and the carrier. The morphology of each component is preserved. 

#### 2.1.6. BET Analysis 

Adsorption analysis with the BET method allows us to compare specific surface areas of commercial HNTs and etched samples. Results are reported in Table 3. The value obtained for commercial HNTs is in good agreement with those reported in the literature [25]. Etching treatments increase specific surface area. This effect is more pronounced in the sample treated with HCl 8M. Higher specific surface areas are beneficial to enhance the active principle adsorption onto halloysite nanotubes.

#### 2.1.7. DSC

**Figure 10:**
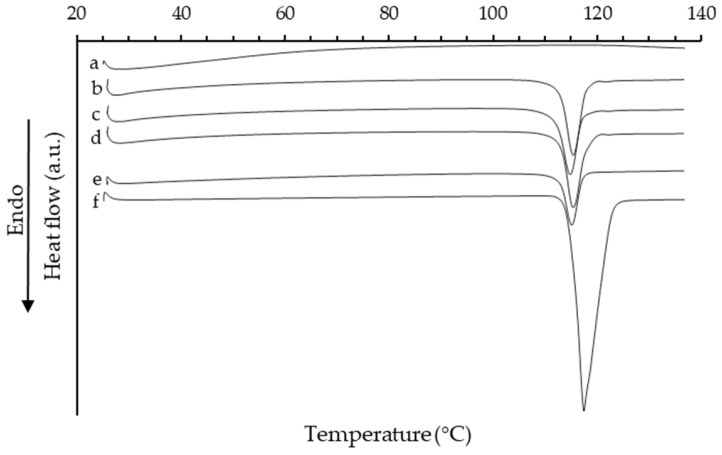
DSC thermograms of H (a), CH (b), CH_HCl_8M (c), CH_H_2_SO_4__0.5M (d), CH_NaOH_0.5M (e), and C (f).

DSC curves are shown in Figure 10. The carvedilol curve shows an endothermic peak with T_onset_ = 115 °C ascribed to the melting process of the crystalline form II [41]. The H sample does not show thermic events. An endothermic effect at 116 °C, due to dehydration, has been reported in the literature [66]; it is not detected in the H sample, as it is anhydrous (see XRPD results). All the curves of the drug–clay systems show the melting peak of carvedilol at the expected temperature value: the drug has been successfully loaded onto the nanoclay carrier and does not modify its physico-chemical properties.

Peak areas have been used to evaluate the melting enthalpy and to assess the amount of crystalline drug loaded onto HNTs (wt%). Results are reported in Table 4. 

#### 2.1.8. Carvedilol Content

Table 5 shows the active principle content (wt%) in drug–clay systems obtained through UV–Vis spectroscopy analysis. All the samples reach good loadings, over 30 wt%, as compared to the theoretical value of 40 wt%. The loading percentages obtained with this technique are higher than those obtained through DSC analysis. This result suggests that carvedilol is loaded on HNTs in both crystalline and amorphous forms. 

#### 2.1.9. Dissolution Tests

**Figure 11:**
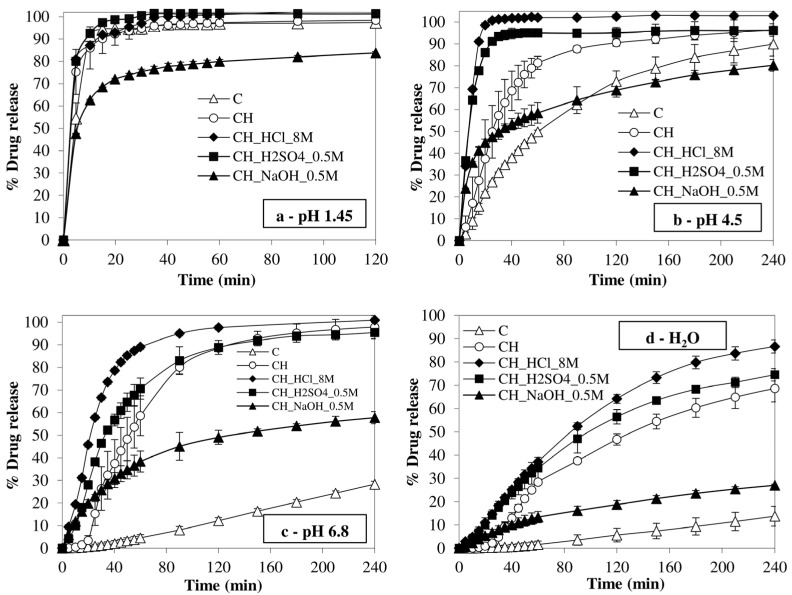
Dissolution profiles of C, CH, CH_HCl_8M, CH_H_2_SO_4__0.5M, and CH_NaOH_0.5M at pH 1.45 (**a**) according to USP monograph, in which the drug is more soluble; at pH 4.5 (**b**), simulating gastric fed conditions; pH 6.8 (**c**), simulating intestinal environment and deionized water (**d**).

Figure 11 shows dissolution profiles of the four samples considered in different conditions simulating the Gastrointestinal (GI) transit: pH 1.45, simulating fasted-state gastric fluid, pH 4.5, fed-state gastric condition, pH 6.8, intestinal environment and deionized water.

The US Pharmacopeia monograph [67] prescribes carrying out the test at pH 1.45 because in these conditions, C is more soluble and has the purpose of being able to carry out quality control of industrial production. As expected, at this pH the drug dissolves in a very short time as well as the hybrids (Figure 11a), except for CH_NaOH_0.5M with alkaline etching that showed a slower dissolution pattern. However, already at pH 4.5 the drug is less soluble, and its dissolution rate is much slower. On the contrary, the hybrid compounds show an improvement in dissolution performances, particularly those with acidic etching (Figure 11b). Even more evident is the improvement of the dissolution characteristics of the HNT hybrid with acidic etching in those media in which the drug is less soluble, i.e., pH 6.8 (Figure 11c), and deionized water (Figure 11d). CH_HCl_8M is always the best sample in terms of dissolution rate and dissolution of the dose in the shortest time. In this way, dissolution performance less dependent on the pH of the medium is obtained.

The dissolution tests were also performed on all the other samples prepared: CH_HCl_2M, CH_HCl_4M, CH_HCl_6M, CH_HCl_12M, and CH_H_2_SO_4__1M. Results are reported in Figure S9.

Relatedly, in these cases, the activation with HCl proved to be very effective in all the conditions considered, while CH_H_2_SO_4__1M seemed less efficient.

The dissolution behavior of the best-performing samples, i.e., CH, CH_HCl_8M, and CH_H_2_SO_4__0.5M samples, was compared with the corresponding physical mixtures (PM) (Figure S10). From the results, it appears that the improvement of the dissolution behavior is due only to the hybridization of the drug because the physical mixtures show a much slower dissolution rate.

#### 2.1.10. Solubility

The equilibrium solubility of these samples was measured in deionized water and at pH 6.8 (Table 6), in which the drug is less soluble. A remarkable increase in the solubility of the hybrid samples is evident, as compared to the drug alone. Again, acid etching proves very effective with a maximum for CH_H_2_SO_4__0.5M, showing two orders of magnitude increase in water.

#### 2.1.11. Contact Angle

**Figure 12:**
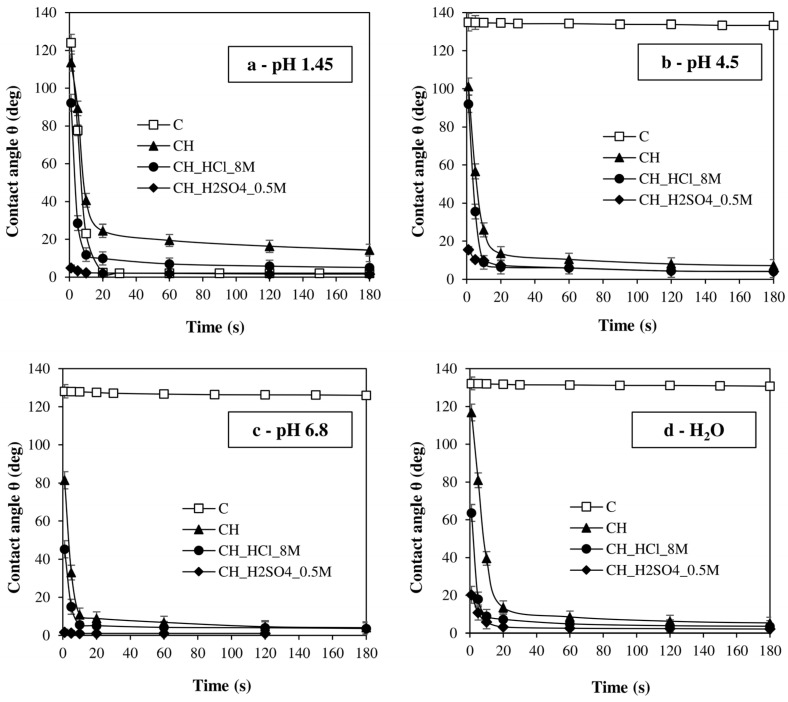
Contact angle (deg) measured on C alone and CH, CH_HCl_8M, and CH_H_2_SO_4__0.5M using different wetting fluids at pH 1.45 (**a**) (USP monograph), in which the drug is more soluble; at pH 4.5 (**b**), simulating gastric fed conditions; pH 6.8 (**c**), simulating intestinal environment and deionized water (**d**).

The contact angle highlights the wettability properties of the samples considered in the different biorelevant fluids (Figure 12).

Carvedilol is only wettable at pH 1.45, while it is very poorly wettable at pH 4.5, 6.8, and in water (contact angle of about 130 degrees constant over time). On the contrary, CH, CH_HCl_8M, and CH_H_2_SO_4__0.5M all have contact angles that tend to zero in less than 10 sec in all the fluids considered. The improved wettability, as well as the increase in solubility, also contribute to the notable improvement in the dissolution performance of these samples.

## 3. Materials and Methods

All the chemicals employed were reagent grade or higher in quality. Halloysite nanotubes (HNTs), hydrochloric acid (HCl), sulphuric acid (H_2_SO_4_), sodium hydroxide (NaOH), and methanol (MeOH) were purchased from Merck (Milano, Italy). Carvedilol (C, C_24_H_26_N_2_O_4_) was kindly donated by Mohes (Barcelona, Spain). 

### 3.1. Synthesis

#### 3.1.1. Acid Activation of HNT with HCl

The acid etching with HCl followed the procedure reported by Wang et al. [68] with some modifications. A total of 1 g of HNT powder was suspended in 10 mL of HCl solution at different concentrations (2, 4, 6, 8, and 12 mol L^−1^) at room temperature. The mixture was mechanically stirred for 4 h and then centrifuged at 6000 rpm for 5 min. The solid was washed with distilled water until pH ≈ 6 was reached. The samples were dried overnight at 105 °C. 

#### 3.1.2. Acid Activation of HNT with H_2_SO_4_

The acid etching with H_2_SO_4_ was performed by following the procedure reported by Surya et al. [69] with some modifications. A total of 1 g of HNT powder was suspended in 10 mL of H_2_SO_4_ solution (0.5 and 1 mol L^−1^). The mixture was mechanically stirred and heated at 70 °C for 2 h and then centrifuged at 6000 rpm for 5 min. The solid was washed with distilled water until pH ≈ 7. The samples were dried overnight at 70 °C. 

#### 3.1.3. Alkaline Activation of HNT with NaOH

The alkaline etching with NaOH was performed by following the procedure reported by Wang et al. [70] with some modifications. A total of 1 g of HNT powder was suspended in 10 mL NaOH solution (0.5 mol L^−1^). The mixture was sonicated at 50 °C for 1 h and then centrifuged at 6000 rpm for 5 min. The solid was washed with distilled water until pH ≈ 7. The samples were dried overnight at 105 °C. 

#### 3.1.4. Carvedilol Loading onto HNT

The loading of carvedilol onto HNT samples was performed by following the incipient wetness impregnation method reported by Li et al. [71]. A concentrated solution of C in methanol was prepared by dissolving 160 mg of active principle in 16 mL of solvent. This solution was added drop by drop to 240 mg of HNT sample, under continuous stirring. The samples were treated overnight at 40 °C in air to completely evaporate the solvent. The carvedilol–halloysite physical mixtures were prepared by weighing the proper amounts (derived from the measured load of the single sample) of the two powders and mixing them in a Turbula apparatus (Bachofen, Basel, Switzerland). 

### 3.2. Characterization Techniques

X-ray powder diffraction (XRPD) measurements were performed with a Bruker D5005 diffractometer (Bruker, Karlsruhe, Germany) equipped with the CuKα tube, curved-graphite monochromator, and scintillation detector. The patterns were collected in the angular range 7° ≤ 2*θ* ≤ 48°, step size of 0.03°, and 5 s/step counting time. A silicon low-background sample holder was used. The samples were fine powders, and no manual grinding was applied.

FT-IR spectra were obtained with a Nicolet FT-IR iS10 Spectrometer (Nicolet, Madison, WI, USA) equipped with ATR (attenuated total reflectance) sampling accessory (Smart iTR with diamond plate) by co-adding 32 scans in the 4000–600 cm^−1^ range at 4 cm^−1^ resolution. 

NMR measurements have been acquired on a 9.4 T Bruker Avance III magnet equipped with a 4 mm MAS probe (MAS speed 10 kHz). The ^13^C spectra were registered using CPMAS protocol with 2500 us contact time, 1k scans, d1 10 s, referred to adamantane signal as secondary standard. The ^27^Al measurements were collected with 120 scans and d1 50 s with single pulse program, referred to a 1M solution of Al(NO_3_)_3_.

SEM measurements were performed using a Zeiss EVO MA10 (Carl Zeiss, Oberkochen, Germany) microscope. The SEM images were collected on gold-sputtered samples in argon atmosphere. Images were obtained at 20 kV voltage at 3 kX and 10 kX magnifications. 

TEM micrographs were taken using a JEOL JEM-1200 EX II (JEOL ltd., Tokyo, Japan) microscope operating at 100 kV high voltage (tungsten filament gun) and equipped with a TEM CCD camera Olympus Mega View III (Olympus soft imaging solutions (OSIS) GmbH, from 2015 EMSIS GmbH, Munster, Germany) with 1376 × 1032 px format. The samples were prepared by drop casting the solution on nickel grids formvar/carbon coated.

The specific surface area of the materials was determined by N_2_ adsorption using the BET method in a Sorptomatic 1990 porosimeter (Thermo Electron, Waltham, MA, USA).

DSC measurements were performed using a DSC Q2000 apparatus interfaced with a data station TA 5000 (TA Instruments, Newcastle, DE, USA). The DSC instrument was calibrated using ultrapure (99.999%) indium (melting point = 156.6 °C; ΔH = 28.54 J g^−1^). The samples were scanned in open aluminum pans at 10 K min^−1^ under nitrogen flow (45 mL min^−1^) in the 20–140° temperature range. 

### 3.3. Carvedilol Content and Dissolution Tests

All the samples were sieved through a 53 µm grid (ASTM Endecotts LTD, London, UK) before all tests.

The drug content of the different samples was measured on diluted solutions in the fluid of maximum solubility, at pH 1.45 with UV–Vis spectroscopy analysis using a Lambda 25 UV spectrophotometer (Perkin-Elmer, Monza, Italia), at 286 nm three replicates.

The dissolution tests were performed following USP guidelines [67], using USP Apparatus 2, paddle (Erweka DT-D6; Erweka, Dusseldorf, Germania). The instrument was set at 37 °C, 50 rpm stirring, and filled with 900 mL of different fluids simulating the gastrointestinal transit:

1. Hydrochloric solution pH 1.45 as required by USP guidelines;
2. Phosphate buffer solution pH 4.5 (fed stomach condition);
3. Phosphate buffer solution pH 6.8 (intestinal condition);
4. Deionized water.

The buffer solutions were prepared as reported in the reagent and buffer solutions section of the USP [72]. The proper amount of the different samples was weighed corresponding to a dose of 6.25 mg of C and it was determined by the loading percentage obtained from the spectrophotometric analysis (16.6 mg for CH, 20.7 mg for CH_HCl_8M, 17.7 mg for CH_H_2_SO_4__0.5M, and 20.4 mg for CH_NaOH_0.5M). During the dissolution tests, samples were taken every five minutes in the first hour of testing and every half an hour after sixty minutes. A peristaltic pump takes the liquid through a filter from the dissolution vessel, transports it to the cells of the UV–Vis spectrophotometer, and then returns it to the vessel. Both pieces of equipment are connected to a computer which guides the whole experiment and acquires the results. 

The results were plotted as the mean of the percent dose released as a function of time with standard deviation (SD). Statistical analysis was performed using the Microsoft Excel statistical tool (MicrosoftOffice 365, MS, Redmond, WA, USA). All the experiments were carried out in triplicate unless otherwise stated.

Finally, to highlight the differences found in the dissolution profiles in terms of release rates, the time to reach 50% of the dose dissolved (td 50%) was determined from the plots (see Supplementary Materials, Table S2).

### 3.4. Solubility

The solubility was measured using the shake flask method: an excess of the sample is placed in a flask and left under continuous magnetic stirring at 200 rpm. At fixed time intervals, a portion of the supernatant is withdrawn and filtered through a 0.45 µm filter (Millipore, Merck, Kenilworth, NJ, USA). The drug content was determined by UV–Vis spectroscopy at 286 nm until reaching a constant value. 

### 3.5. Contact Angle

The wettability of the pure active, C, and the hybrid compounds, CH, CH_HCl_8M, and CH_H_2_SO_4__0.5M, was determined using a Contact Angle Meter DMe-211Plus (NTG Nuova Tecnogalenica, Cernusco, I). A 10 µL drop of deionized water, hydrochloric acid solution pH 1.45, and phosphate buffers pH 4.5 or pH 6.8 was dropped from a needle onto the surface of the samples. Their images were acquired in progressive times (from t = 0 s up to 180 s). The contact angles were measured by a suitable software provided by the equipment. The test was repeated three times for each sample.

## 4. Conclusions

In this work, new carvedilol-etched halloysite composites were synthesized by a simple and viable impregnation method. The results obtained by several characterization techniques put into evidence carvedilol is successfully loaded in the 30–37% weight amount, depending on the sample. The drug and carrier structures are maintained and the particles of the two components are in intimate contact, as evidenced by TEM. The carvedilol molecules do not interact with the aluminum internal surface of the HNTs, as demonstrated by ^27^Al solid-state NMR. Indeed, ^13^C solid-state NMR and FT-IR demonstrate that the aliphatic portion, the functional groups, and, for inductive effects, the aromatic carbons near the functional groups are involved in interactions with the halloysite component and/or with other carvedilol moieties.

All the investigated composites display improved dissolution rates and enhanced wettability and solubility, as compared to the carvedilol. Indeed, the best performance is obtained for the carvedilol and halloysite etched with HCl 8M; this sample also exhibits the highest specific surface area (91 m^2^ g^−1^). 

The clay nanomaterials are non-toxic, cost-effective, highly biocompatible, and may be easily functionalized to enable selective inside/outside drug loading with specific targeting.

The hybridization of this drug allowed a normalization of its dissolution characteristics. This process makes the passage of the drug into solution independent of the environmental conditions of the gastrointestinal tract and makes its absorption less variable and more predictable. In this way its absorption performances do not depend on the presence of food and could be administered at any time of the day. 

The next step would be to verify the in vivo bioavailability of the most promising carvedilol–halloysite hybrid and to study its possible use in a therapeutic application. Moreover, possible loading into the HNTs lumen could be investigated through functionalization of the inner surface to make it more reactive toward carvedilol. 

## Figures and Tables

**Table 1:** List and names of the investigated samples.

ALPHANUMERIC CODE	API	HNT
H	-	Commercial
H_HCl_2M	-	Etching with HCl 2M
H_HCl_4M	-	Etching with HCl 4M
H_HCl_6M	-	Etching with HCl 6M
H_HCl_8M	-	Etching with HCl 8M
H_HCl_12M	-	Etching with HCl 12M
H_H_2_SO_4__0.5M	-	Etching with H_2_SO_4_ 0.5M
H_H_2_SO_4__1M	-	Etching with H_2_SO_4_ 1M
H_NaOH_0.5M	-	Etching with NaOH 0.5M
C	Carvedilol	-
CH	Carvedilol	Commercial
CH_HCl_2M	Carvedilol	Etching with HCl 2M
CH_HCl_4M	Carvedilol	Etching with HCl 4M
CH_HCl_6M	Carvedilol	Etching with HCl 6M
CH_HCl_8M	Carvedilol	Etching with HCl 8M
CH_HCl_12M	Carvedilol	Etching with HCl 12M
CH_H_2_SO_4__0.5M	Carvedilol	Etching with H_2_SO_4_ 0.5M
CH_H_2_SO_4__1M	Carvedilol	Etching with H_2_SO_4_ 1M
CH_NaOH_0.5M	Carvedilol	Etching with NaOH 0.5M
**CARVEDILOL–HNT PHYSICAL MIXTURES**		
CH_PM	Carvedilol	Commercial
CH_HCl_8M_PM	Carvedilol	Etching with HCl 8M
CH_H_2_SO_4__0.5M_PM	Carvedilol	Etching with H_2_SO_4_ 0.5M

**Table 2:** Comparison of the IR bands for active principle, physical mixture, and active principle–halloysite hybrids.

Carbon	Bond	Carvedilol	CH_HCl_8M_PM ^(a)^	CH_HCl_8M ^(b)^
C1, C11	C-O	1097 cm^−1^	1097 cm^−1^	1087 cm^−1^
C2, C7, C13	Aromatic ring	1444 cm^−1^ 1499 cm^−1^ 1606 cm^−1^ 1630 cm^−1^	1444 cm^−1^ 1499 cm^−1^ 1606 cm^−1^ 1630 cm^−1^	1443 cm^−1^ 1501 cm^−1^ 1608 cm^−1^ very weak
C9, C10	Stretching C-N	1211 cm^−1^ 1250 cm^−1^	1211 cm^−1^ 1250 cm^−1^	1214 cm^−1^ 1256 cm^−1^

^(a)^ Chosen as an example of the PM system: the same band positions are detected in other PM samples. ^(b)^ Chosen as an example of the CH hybrid system: the same band positions are detected in other CH hybrid samples.

**Table 3:** Specific surface area of modified and unmodified halloysite samples.

Sample	Specific Surface Area (m^2^ g^−1^)
H	58.20
H_HCl_8M	97.92
H_H_2_SO_4__0.5M	79.68
H_NaOH_0.5M	71.87

**Table 4:** DSC analysis data.

Sample	T_onset_ (°C)	ΔH_fus_ (J g^−1^)	Carvedilol Content (wt%)
C	115	117.60	100
CH	113	33.29	28.3
CH_HCl_8M	112	26.99	23.0
CH_H_2_SO_4__0.5M	113	29.19	24.8
CH_NaOH_0.5M	113	22.10	18.8

**Table 5:** Carvedilol content (wt%) determined by UV–Vis spectroscopy.

Sample	Carvedilol Content (wt%)
CH	37.6
CH_HCl_8M	30.2
CH_H_2_SO_4__0.5M	35.3
CH_NaOH_0.5M	30.6

**Table 6:** Equilibrium solubility at room temperature of samples C, CH, CH_HCl_8M, and CH_H_2_SO_4__0.5M.

Equilibrium Solubility at 21 °C (mg/L)				
Fluids	C	CH	CH_HCl_8M	CH_H_2_SO_4__0.5M
Deionized water	5.4 ± 0.1	14.2 ± 1.2	29.6 ± 1.6	260.5 ± 4.2
pH 6.8 buffer	17.7 ± 0.6	31.2 ± 2.8	42.4 ± 2.3	32.1 ± 0.9

## Data Availability

The data presented in this study are available on request from the corresponding author.

## Supplementary Materials

The following supporting information can be downloaded at: https://www.mdpi.com/article/10.3390/molecules28083405/s1, Figures S1–S3: XRPD patterns of etched HNT, carvedilol–HNT composites and carvedilol–HNT physical mixtures; Figure S4: The ^27^Al solid-state NMR spectra of HNT and carvedilol–HNT composites; Figures S5–S7: FT-IR spectra of etched HNT, carvedilol–HNT composites, and carvedilol–HNT physical mixtures; Figure S8: SEM images of carvedilol–HNT physical mixtures; Figures S9 and S10: dissolution profiles at pH values of 1.45, 4.5, 6.8, and in deionized water; Table S1: FT-IR bands of HNT, carvedilol, carvedilol–HNT composites, and carvedilol–HNT physical mixtures. Table S2: Comparison of the time required to deliver 50% of the dose from all samples in pH 1.45, pH 4.5, pH 6.8, and deionized water.

## References

[1] Good D.J., Rodríguez-Hornedo N. (2009). Solubility Advantage of Pharmaceutical Cocrystals. Cryst. Growth Des..

[2] Thayer J.F., Yamamoto S.S., Brosschot J.F. (2010). The Relationship of Autonomic Imbalance, Heart Rate Variability and Cardiovascular Disease Risk Factors. Int. J. Cardiol..

[3] Kumar S., Singh P. (2016). Various Techniques for Solubility Enhancement: An Overview. Pharma Innov..

[4] Braga D., Grepioni F., Maini L., Polito M., Hosseini M.W. (2009). Crystal Polymorphism and Multiple Crystal Forms. Molecular Networks.

[5] Lu J., Li Z., Jiang X. (2010). Polymorphism of Pharmaceutical Molecules: Perspectives on Nucleation. Front. Chem. Sci. Eng..

[6] DeMatos L.L., Williams A.C., Booth S.W., Petts C.R., Taylor D.J., Blagden N. (2007). Solvent Influences on Metastable Polymorph Lifetimes: Real-Time Interconversions Using Energy Dispersive X-Ray Diffractometry. J. Pharm. Sci..

[7] Karki S., Friscić T., Jones W., Motherwell W.D.S. (2007). Screening for Pharmaceutical Cocrystal Hydrates via Neat and Liquid-Assisted Grinding. Mol. Pharm..

[8] Pudipeddi M., Serajuddin A.T.M. (2005). Trends in Solubility of Polymorphs. J. Pharm. Sci..

[9] Braga D., Chelazzi L., Grepioni F., Dichiarante E., Chierotti M., Gobetto R. (2013). Molecular Salts of Anesthetic Lidocaine with Dicarboxylic Acids: Solid-State Properties and a Combined Structural and Spectroscopic Study. Cryst. Growth Des..

[10] Bruni G., Maietta M., Maggi L., Bini M., Capsoni D., Ferrari S., Boiocchi M., Berbenni V., Milanese C., Marini A. (2012). Perphenazine–Fumaric Acid Salts with Improved Solubility: Preparation, Physico-Chemical Characterization and in Vitro Dissolution. CrystEngComm.

[11] Trask A.V., Haynes D.A., Motherwell W.D.S., Jones W. (2006). Screening for Crystalline Salts via Mechanochemistry. Chem. Commun..

[12] Bethune S.J., Huang N., Jayasankar A., Rodríguez-Hornedo N. (2009). Understanding and Predicting the Effect of Cocrystal Components and PH on Cocrystal Solubility. Cryst. Growth Des..

[13] Bruni G., Maietta M., Maggi L., Mustarelli P., Ferrara C., Berbenni V., Milanese C., Girella A., Marini A. (2013). Preparation and Physicochemical Characterization of Acyclovir Cocrystals with Improved Dissolution Properties. J. Pharm. Sci..

[14] Friščić T., Jones W. (2007). Cocrystal Architecture and Properties: Design and Building of Chiral and Racemic Structures by Solid–Solid Reactions. Faraday Discuss..

[15] Jirát J., Ondo D., Babor M., Ridvan L., Šoóš M. (2019). Complex Methodology for Rational Design of Apremilast-Benzoic Acid Co-Crystallization Process. Int. J. Pharm..

[16] Loftsson T. (2017). Drug Solubilization by Complexation. Int. J. Pharm..

[17] Li Q., Li X., Zhao C. (2020). Strategies to Obtain Encapsulation and Controlled Release of Small Hydrophilic Molecules. Front. Bioeng. Biotechnol..

[18] Tran T.T.D., Tran P.H.L. (2019). Nanoconjugation and Encapsulation Strategies for Improving Drug Delivery and Therapeutic Efficacy of Poorly Water-Soluble Drugs. Pharmaceutics.

[19] Yang K.Y., Hwang D.H., Yousaf A.M., Kim D.-W., Shin Y.-J., Bae O.-N., Kim Y.-I., Kim J.O., Yong C.S., Choi H.-G. (2013). Silymarin-Loaded Solid Nanoparticles Provide Excellent Hepatic Protection: Physicochemical Characterization and in Vivo Evaluation. Int. J. Nanomed..

[20] Massaro M., Colletti C.G., Lazzara G., Riela S. (2018). The Use of Some Clay Minerals as Natural Resources for Drug Carrier Applications. J. Funct. Biomater..

[21] Abdullayev E., Lvov Y. (2013). Halloysite Clay Nanotubes as a Ceramic “Skeleton” for Functional Biopolymer Composites with Sustained Drug Release. J. Mater. Chem. B.

[22] Yu L., Wang H., Zhang B., Liu J. (2015). Recent Advance of Halloysite Nanotubes Derived Composites in Water Treatment. Environ. Sci. Nano.

[23] Fizir M., Dramou P., Dahiru N.S., Ruya W., Huang T., He H. (2018). Halloysite Nanotubes in Analytical Sciences and in Drug Delivery: A Review. Mikrochim Acta.

[24] Yuan P., Tan D., Annabi-Bergaya F. (2015). Properties and Applications of Halloysite Nanotubes: Recent Research Advances and Future Prospects. Appl. Clay Sci..

[25] Lvov Y., Aerov A., Fakhrullin R. (2014). Clay Nanotube Encapsulation for Functional Biocomposites. Adv. Colloid Interface Sci..

[26] Liu T., Zhang J., Ouyang P., Fu L., Yang H. (2021). The Relation between Nanotube Diameter, Length and Surface Area and Pore Volume of Multi-Walled Spiral Halloysite Nanotubes: A Theoretical Study. Appl. Clay Sci..

[27] Peixoto A.F., Fernandes A.C., Pereira C., Pires J., Freire C. (2016). Physicochemical Characterization of Organosilylated Halloysite Clay Nanotubes. Microporous Mesoporous Mater..

[28] Yang J., Wu Y., Shen Y., Zhou C., Li Y.-F., He R.-R., Liu M. (2016). Enhanced Therapeutic Efficacy of Doxorubicin for Breast Cancer Using Chitosan Oligosaccharide-Modified Halloysite Nanotubes. ACS Appl. Mater. Interfaces.

[29] Abdullayev E., Joshi A., Wei W., Zhao Y., Lvov Y. (2012). Enlargement of Halloysite Clay Nanotube Lumen by Selective Etching of Aluminum Oxide. ACS Nano.

[30] White R.D., Bavykin D.V., Walsh F.C. (2012). The Stability of Halloysite Nanotubes in Acidic and Alkaline Aqueous Suspensions. Nanotechnology.

[31] Horváth E., Kristóf J., Kurdi R., Makó É., Khunová V. (2011). Study of Urea Intercalation into Halloysite by Thermoanalytical and Spectroscopic Techniques. J. Therm. Anal. Calorim..

[32] Nicolini K.P., Fukamachi C.R.B., Wypych F., Mangrich A.S. (2009). Dehydrated Halloysite Intercalated Mechanochemically with Urea: Thermal Behavior and Structural Aspects. J. Colloid Interface Sci..

[33] Yah W.O., Takahara A., Lvov Y.M. (2012). Selective Modification of Halloysite Lumen with Octadecylphosphonic Acid: New Inorganic Tubular Micelle. J. Am. Chem. Soc..

[34] Yah W.O., Xu H., Soejima H., Ma W., Lvov Y., Takahara A. (2012). Biomimetic Dopamine Derivative for Selective Polymer Modification of Halloysite Nanotube Lumen. J. Am. Chem. Soc..

[35] Ahmed F.R., Shoaib M.H., Azhar M., Um S.H., Yousuf R.I., Hashmi S., Dar A. (2015). In-Vitro Assessment of Cytotoxicity of Halloysite Nanotubes against HepG2, HCT116 and Human Peripheral Blood Lymphocytes. Colloids Surf. B Biointerfaces.

[36] Capsoni D., Lucini P., Conti D.M., Bianchi M., Maraschi F., De Felice B., Bruni G., Abdolrahimi M., Peddis D., Parolini M. (2022). Fe_3_O_4_-Halloysite Nanotube Composites as Sustainable Adsorbents: Efficiency in Ofloxacin Removal from Polluted Waters and Ecotoxicity. Nanomaterials.

[37] Frishman W.H. (1998). Carvedilol. N. Engl. J. Med..

[38] Packer M., Bristow M.R., Cohn J.N., Colucci W.S., Fowler M.B., Gilbert E.M., Shusterman N.H. (1996). The Effect of Carvedilol on Morbidity and Mortality in Patients with Chronic Heart Failure. U.S. Carvedilol Heart Failure Study Group. N. Engl. J. Med..

[39] Loftsson T., Vogensen S.B., Desbos C., Jansook P. (2008). Carvedilol: Solubilization and Cyclodextrin Complexation: A Technical Note. Aaps Pharmscitech.

[40] Sip S., Paczkowska-Walendowska M., Rosiak N., Miklaszewski A., Grabańska-Martyńska K., Samarzewska K., Cielecka-Piontek J. (2021). Chitosan as Valuable Excipient for Oral and Topical Carvedilol Delivery Systems. Pharmaceuticals.

[41] Beattie K., Phadke G., Novakovic J., Brittain H.G. (2013). Chapter Four—Carvedilol. Profiles of Drug Substances, Excipients and Related Methodology.

[42] Ha E.-S., Kim J.-S., Lee S.-K., Sim W.-Y., Jeong J.-S., Kim M.-S. (2019). Equilibrium Solubility and Solute-Solvent Interactions of Carvedilol (Form I) in Twelve Mono Solvents and Its Application for Supercritical Antisolvent Precipitation. J. Mol. Liq..

[43] Shete A., Yadav A., Muppavarapu S. (2012). Chitosan and Chitosan Chlorhydrate Based Various Approaches for Enhancement of Dissolution Rate of Carvedilol. Daru J. Fac. Pharm. Tehran Univ. Med Sci..

[44] Liu D., Xu H., Tian B., Yuan K., Pan H., Ma S., Yang X., Pan W. (2012). Fabrication of Carvedilol Nanosuspensions through the Anti-Solvent Precipitation-Ultrasonication Method for the Improvement of Dissolution Rate and Oral Bioavailability. Aaps Pharmscitech.

[45] Lee J. (2013). Adipose Tissue Macrophages in the Development of Obesity-Induced Inflammation, Insulin Resistance and Type 2 Diabetes. Arch. Pharm. Res..

[46] Eesam S., Bhandaru J.S., Naliganti C., Bobbala R.K., Akkinepally R.R. (2020). Solubility Enhancement of Carvedilol Using Drug–Drug Cocrystallization with Hydrochlorothiazide. Future J. Pharm. Sci..

[47] Levis S.R., Deasy P.B. (2002). Characterisation of Halloysite for Use as a Microtubular Drug Delivery System. Int. J. Pharm..

[48] Aytekin M.T., Hoşgün H.L. (2020). Characterization Studies of Heat-Treated Halloysite Nanotubes. Chem. Pap..

[49] Lvov Y. (2013). Functional Polymer–Clay Nanotube Composites with Sustained Release of Chemical Agents. Prog. Polym. Sci..

[50] Tian X., Wang W., Tian N., Zhou C., Yang C., Komarneni S. (2016). Cr(VI) Reduction and Immobilization by Novel Carbonaceous Modified Magnetic Fe3O4/Halloysite Nanohybrid. J. Hazard. Mater..

[51] Xie Y., Qian D., Wu D., Ma X. (2011). Magnetic Halloysite Nanotubes/Iron Oxide Composites for the Adsorption of Dyes. Chem. Eng. J..

[52] Prado L.D., Rocha H.V.A., Resende J.A.L.C., Ferreira G.B., de Figuereido Teixeira A.M.R. (2014). An Insight into Carvedilol Solid Forms: Effect of Supramolecular Interactions on the Dissolution Profiles. CrystEngComm.

[53] Zheng P., Du Y., Chang P.R., Ma X. (2015). Amylose–Halloysite–TiO_2_ Composites: Preparation, Characterization and Photodegradation. Appl. Surf. Sci..

[54] Amjadi M., Samadi A., Manzoori J.L. (2015). A Composite Prepared from Halloysite Nanotubes and Magnetite (Fe_3_O_4_) as a New Magnetic Sorbent for the Preconcentration of Cadmium(II) Prior to Its Determination by Flame Atomic Absorption Spectrometry. Microchim. Acta.

[55] Cheng H., Frost R.L., Yang J., Liu Q., He J. (2010). Infrared and Infrared Emission Spectroscopic Study of Typical Chinese Kaolinite and Halloysite. Spectrochim. Acta Part A Mol. Biomol. Spectrosc..

[56] Gadape H., Parikh K. (2011). Quantitative Determination and Validation of Carvedilol in Pharmaceuticals Using Quantitative Nuclear Magnetic Resonance Spectroscopy. Anal. Methods.

[57] Wen X., Tan F., Jing Z., Liu Z. (2004). Preparation and Study the 1:2 Inclusion Complex of Carvedilol with β-Cyclodextrin. J. Pharm. Biomed. Anal..

[58] Zielińska-Pisklak M.A., Pisklak D.M., Wawer I. (2011). 1H and 13C NMR Characteristics of β-Blockers. Magn. Reson. Chem..

[59] Ravikumar A.A., Kulkarni P.K., Osmani R.A.M., Hani U., Ghazwani M., Fatease A.A., Alamri A.H., Gowda D.V. (2022). Carvedilol Precipitation Inhibition by the Incorporation of Polymeric Precipitation Inhibitors Using a Stable Amorphous Solid Dispersion Approach: Formulation, Characterization, and In Vitro In Vivo Evaluation. Polymers.

[60] Bruni G., Maggi L., Mustarelli P., Sakaj M., Friuli V., Ferrara C., Berbenni V., Girella A., Milanese C., Marini A. (2019). Enhancing the Pharmaceutical Behavior of Nateglinide by Cocrystallization: Physicochemical Assessment of Cocrystal Formation and Informed Use of Differential Scanning Calorimetry for Its Quantitative Characterization. J. Pharm. Sci..

[61] Bruni G., Monteforte F., Maggi L., Friuli V., Ferrara C., Mustarelli P., Girella A., Berbenni V., Capsoni D., Milanese C. (2020). Probenecid and Benzamide: Cocrystal Prepared by a Green Method and Its Physico-Chemical and Pharmaceutical Characterization. J. Therm. Anal. Calorim..

[62] Maggi L., Bruni G., Ferrara C., Puscalau C., Quinzeni I., Friuli V., Monteforte F., Capsoni D. (2023). Zaltoprofen-Layered Double Hydroxide Hybrids to Enhance Zaltoprofen Solubility and Dissolution Rate. Appl. Clay Sci..

[63] He H., Ji S., Tao Q., Zhu J., Chen T., Liang X., Li Z., Dong H. (2017). Transformation of Halloysite and Kaolinite into Beidellite under Hydrothermal Condition. Am. Mineral..

[64] Ferrara C., Tealdi C., Pedone A., Menziani M.C., Rossini A.J., Pintacuda G., Mustarelli P. (2013). Local versus Average Structure in LaSrAl3O7: A NMR and DFT Investigation. J. Phys. Chem. C.

[65] MacKenzie K., Smith M.E. (2012). Multinuclear Solid-State Nuclear Magnetic Resonance of Inorganic Materials.

[66] Gao M., Lu L., Wang X., Lin H., Zhou Q. (2017). Preparation of a Novel Breviscapine-Loaded Halloysite Nanotubes Complex for Controlled Release of Breviscapine. IOP Conf. Ser. Mater. Sci. Eng..

[67] (2017). Carvedilol Tablets/Official Monographs. The United States Pharmacopoeia (USP40-NF35).

[68] Wang Q., Zhang J., Zheng Y., Wang A. (2014). Adsorption and Release of Ofloxacin from Acid- and Heat-Treated Halloysite. Colloids Surf. B Biointerfaces.

[69] Surya I., Waesateh K., Masa A., Hayeemasae N. (2021). Selectively Etched Halloysite Nanotubes as Performance Booster of Epoxidized Natural Rubber Composites. Polymers.

[70] Wang Q., Zhang J., Wang A. (2013). Alkali Activation of Halloysite for Adsorption and Release of Ofloxacin. Appl. Surf. Sci..

[71] Li Y., Wang T., Wang J., Jiang T., Cheng G., Wang S. (2011). Functional and Unmodified MWNTs for Delivery of the Water-Insoluble Drug Carvedilol—A Drug-Loading Mechanism. Appl. Surf. Sci..

[72] (2017). Reagents: Solutions/Buffer Solutions 2017. The United States Pharmacopeia (USP40-NF35).

