# Supplementary material for: Synthesis and Characterization of Carvedilol-Etched Halloysite Nanotubes Composites with Enhanced Drug Solubility and Dissolution Rate
Source: Molecules. 2023 Apr 12;28(8):3405. doi: 10.3390/molecules28083405 (PMC10142978; doi:10.3390/molecules28083405)
Supplement: Supplementary file 1 [file molecules-28-03405-s001.zip › molecules-2317180-supplementary.docx]

Synthesis and Characterization of Carvedilol-Etched Halloysite Nanotubes Composites with Enhanced Drug Solubility and Dissolution Rate

Lauretta Maggi ^1^, Claudia Urru ^2^, Valeria Friuli ^1^, Chiara Ferrara ^3^, Debora Maria Conti ^2^, Giovanna Bruni ^2^, and Doretta Capsoni ^2,^*

^1^ Department of Drug Sciences, University of Pavia, Via Taramelli 12, 27100 Pavia, Italy; [lauretta.maggi@unipv.it](mailto:lauretta.maggi@unipv.it) (L.M.); [valeria.friuli@unipv.it](mailto:valeria.friuli@unipv.it) (V.F.)

^2^ Department of Chemistry, Physical Chemistry Section & C.S.G.I. (Consorzio Interuniversitario per lo Sviluppo dei Sistemi a Grande Interfase), University of Pavia, 27100 Pavia, Italy; [claudia.urru01@universitadipavia.it](mailto:claudia.urru01@universitadipavia.it) (C.U.); [deboramaria.conti01@universitadipavia.it](mailto:deboramaria.conti01@universitadipavia.it) (D.M.C.); giovanna.bruni@unipv.it (G.B.)

^3^ Department of Materials Science, University of Milano-Bicocca, via Cozzi 55, 20125 Milano, Italy; [chiara.ferrara@unimib.it](mailto:chiara.ferrara@unimib.it) (C.F.)

***** Correspondence: doretta.capsoni@unipv.it (D.C.) Tel.: +39-0382-987213


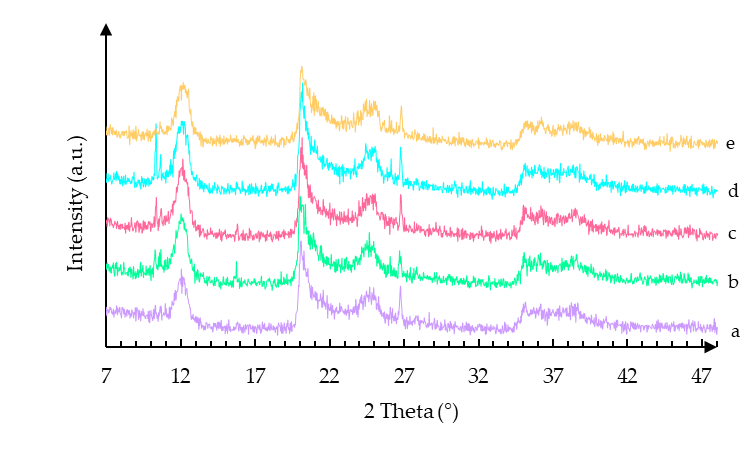


**Figure S1**. XRPD pattern of H_HCl_2M (a), H_HCl_4M (b), H_HCl_6M (c), H_HCl_12M (d), and H_H_2_SO_4__1M (e).


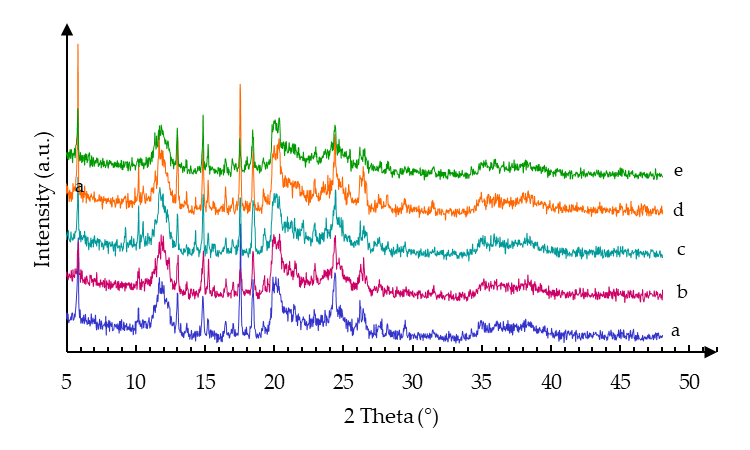


**Figure S2**. XRPD pattern of CH_HCl_2M (a), CH_HCl_4M (b), CH_HCl_6M (c), CH_HCl_12M (d), and CH_H_2_SO_4__1M (e).


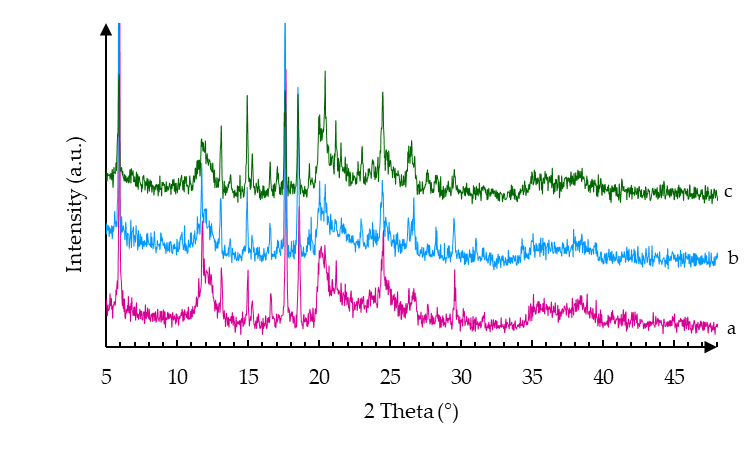


**Figure S3.** XRPD pattern of CH­_PM, (a) CH_HCl_8M_PM (b), and CH_H_2_SO_4__0.5M_PM (c).


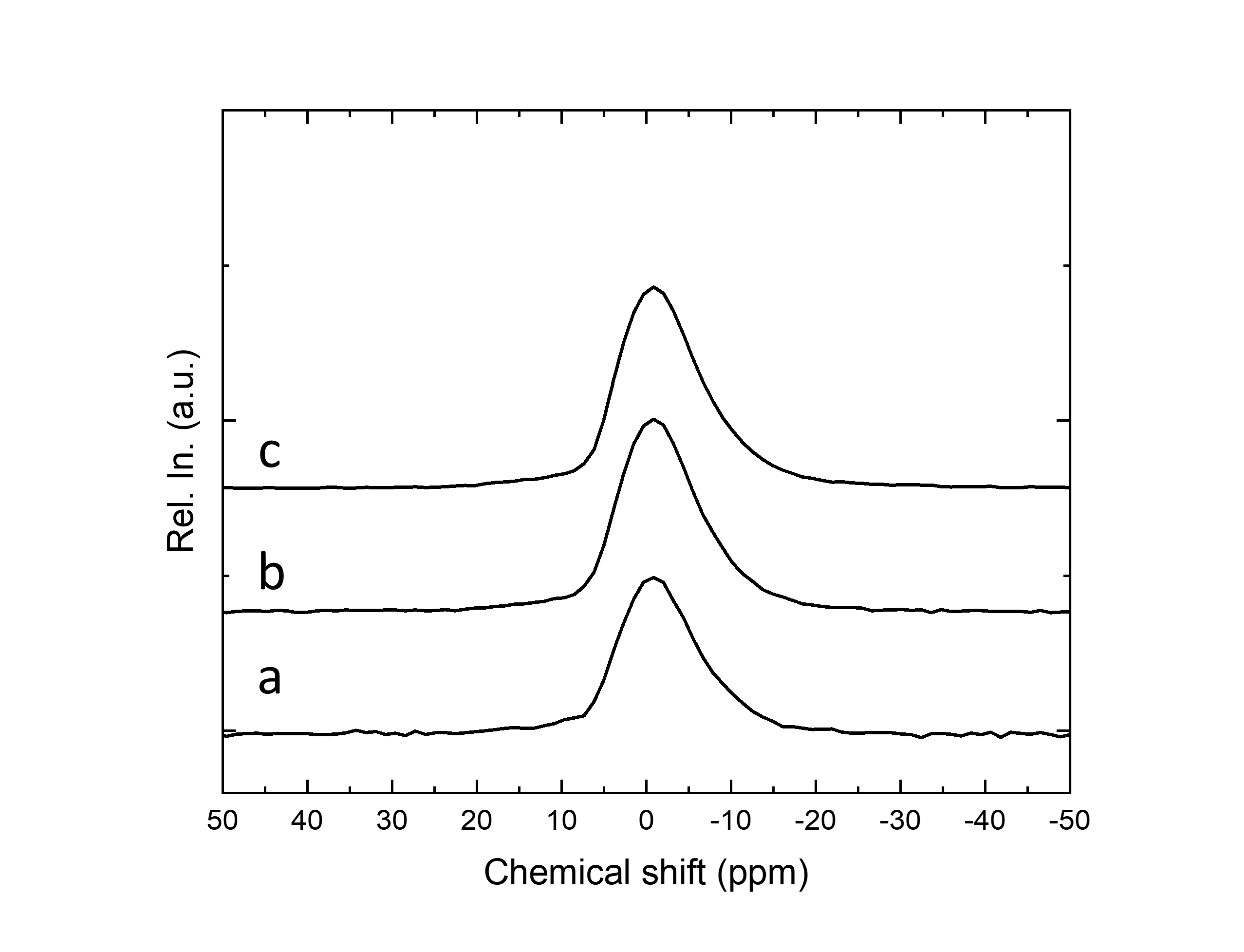


**Figure S4.** ^27^Al spectra for the CH_H_2_SO_4__0.5M (a), CH_HCl_8M (b), and H (c) samples.


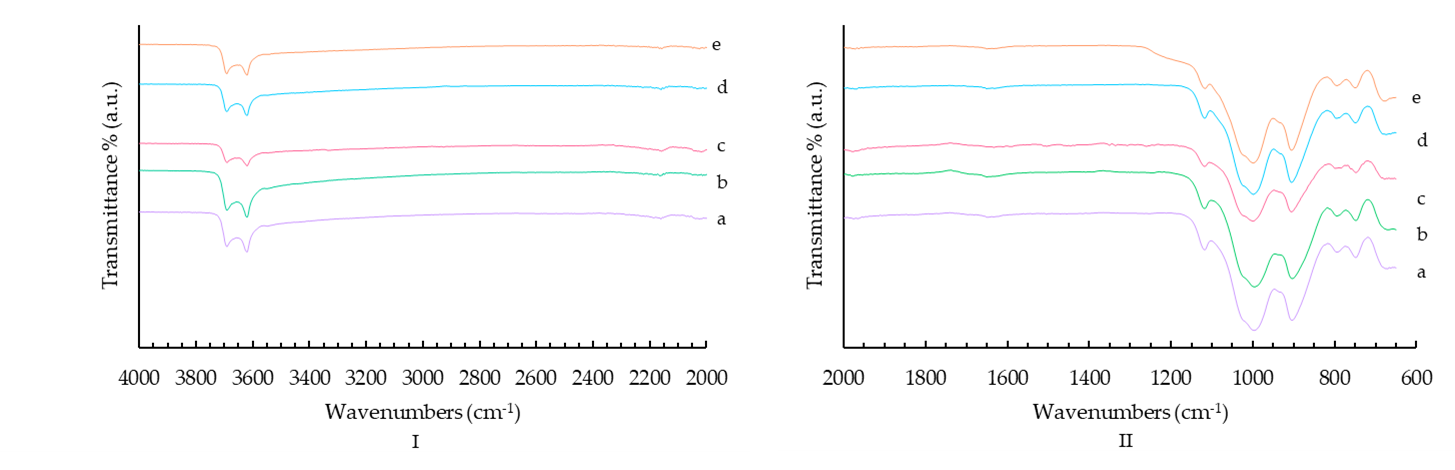


**Figure S5.** FT-IR spectra of H_HCl_2M (a), H_HCl_4M (b), H_HCl_6M (c), H_HCl_12M (d) and H_H_2_SO_4__1M (e) in 4000 – 2000 cm^-1^ (I) and 2000 – 600 cm^-1^ (II) wavenumber range.


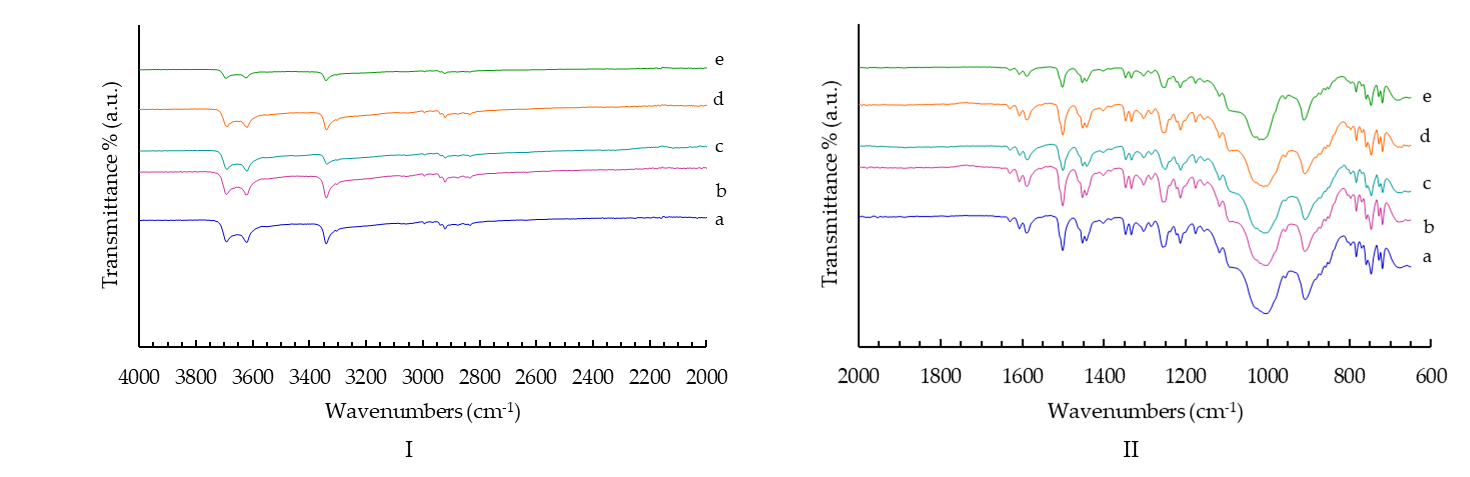


**Figure S6.** FT-IR spectra of CH_HCl_2M (a), CH_HCl_4M (b), CH_HCl_6M (c), CH_HCl_12M (d), and CH_H_2_SO_4__1M (e) in 4000 – 2000 cm^-1^ (I) and 2000 – 600 cm^-1^ (II) wavenumber range.


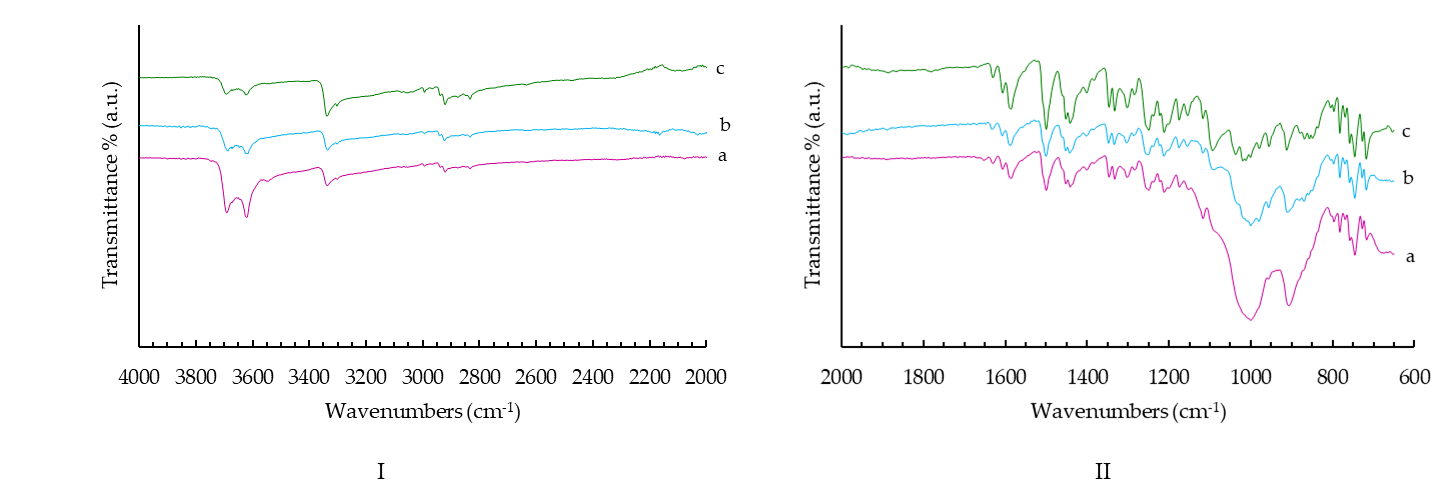


**Figure S7.** FT-IR spectra of CH­_PM, (a) CH_HCl_8M_PM (b), and CH_H_2_SO_4__0.5M_PM (c) in 4000 – 2000 cm^-1^ (I) and 2000 – 600 cm^-1^ (II) wavenumber range.


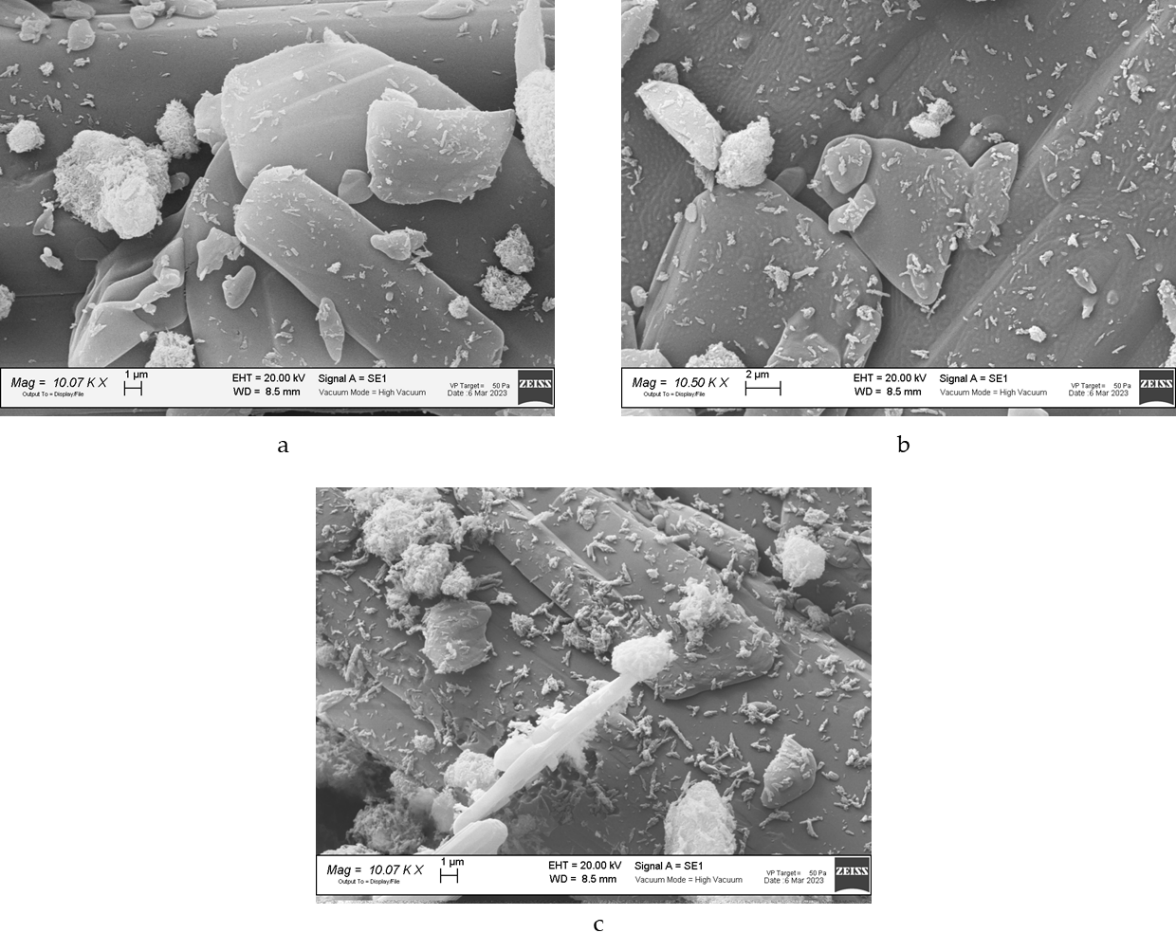


**Figure S8.** SEM images at 10 kX magnification of CH_PM (a), CH_HCl_8M (b), and CH_H_2_SO_4__0.5M (c).


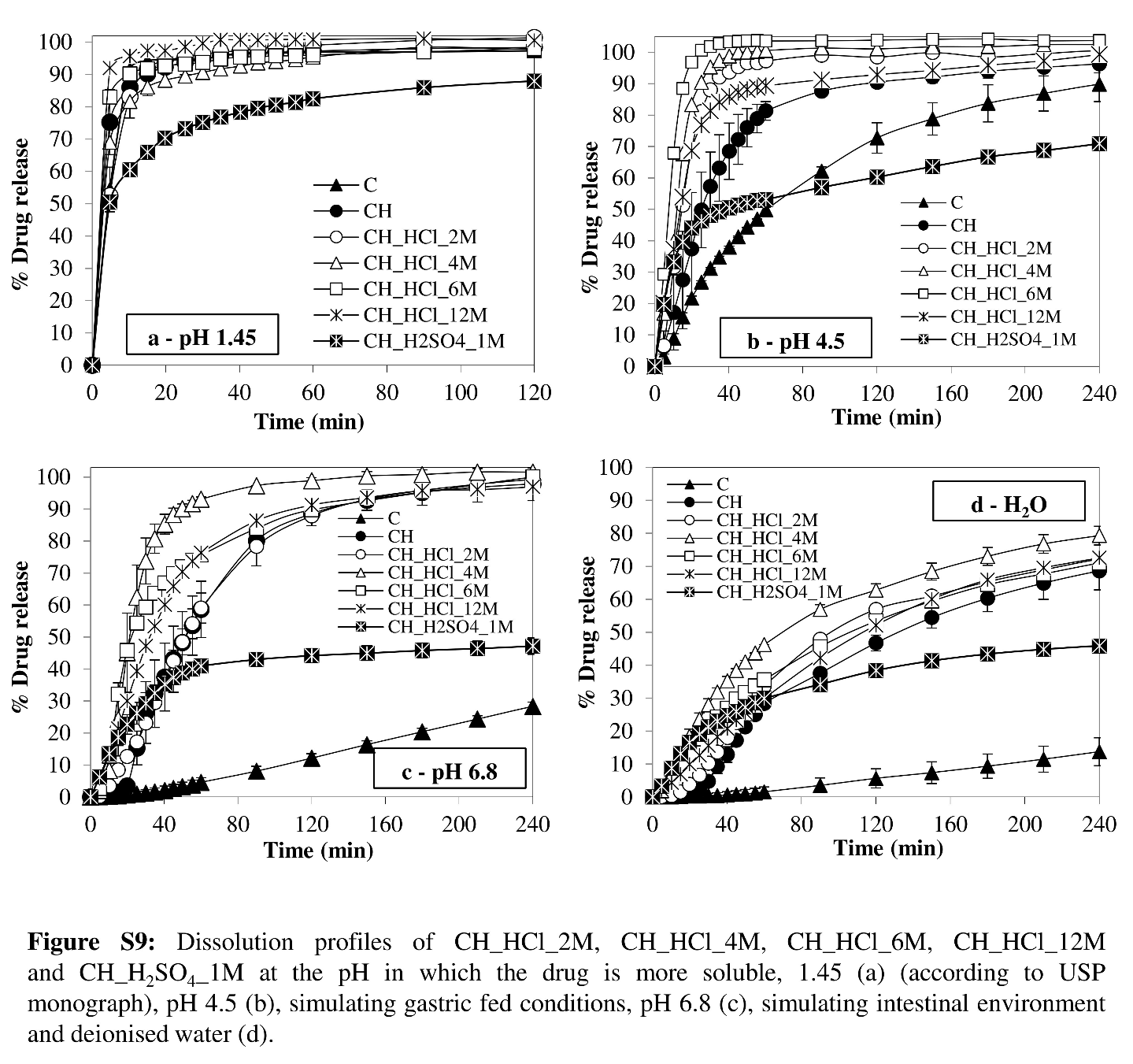


**Figure S9.** Dssolution profiles of CH_HCl_2M, CH_HCl_4M, CH_HCl_6M, CH_HCl_12M and CH_H2SO4_1M at the pH in which the drug is more soluble, 1.45 (according to USP monograph), pH 4.5, simulating gastric fed conditions, pH 6.8, simulating intestinal environment and deionized water.


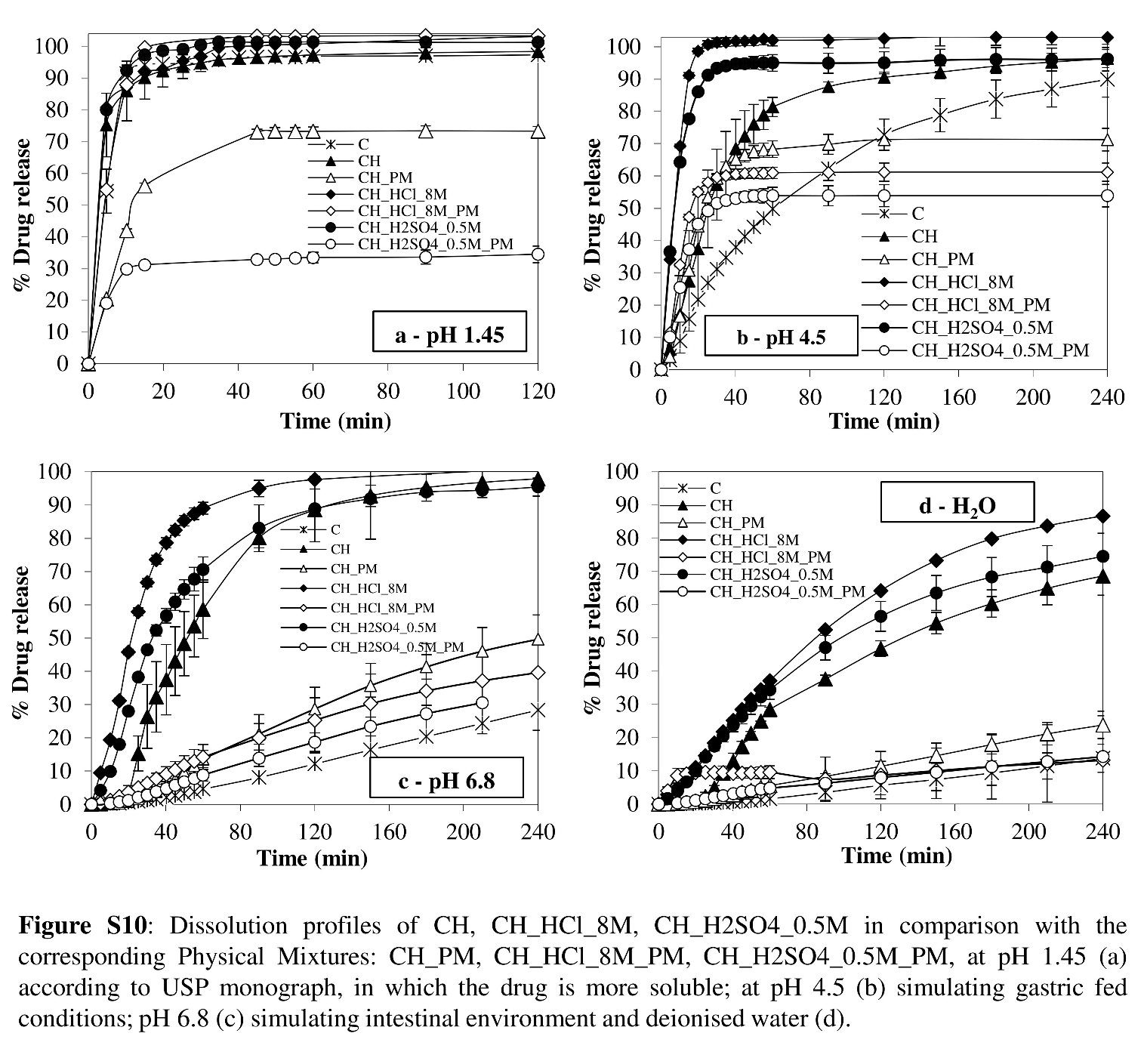


**Figure S10.** Dissolution profiles of CH, CH_HCl_8M, CH_H2SO4_0.5M in comparison with the corresponding Physical Mixtures: CH_PM, CH_HCl_8M_PM, CH_H2SO4_0.5M_PM, at pH 1.45 (a) according to USP monograph, in which the drug is more soluble; at pH 4.5 (b) simulating gastric fed conditions; pH 6.8 (c) simulating intestinal environment and deionised water (d).

**Table S1.** FT-IR bands and assignments.

| **Assignments** | **Commercial H**  **Positions (cm^-1^)** | **Etched H**  **Positions (cm^-1^)** | **Carvedilol**  **Positions (cm^-1^)** | **CH systems**  **Positions (cm^-1^)** |
| --- | --- | --- | --- | --- |
| **Sample** | **H** | **H_HCl_8M** | **C** | **CH_HCl_8M** |
| Al-OH stretching of inner surface groups | 3691  3619 | 3691  3619 | - | 3693  3623 |
| O-H stretching of water | 3545 | - | - | - |
| O-H stretching vibration | - | - | 3335 | 3340 |
| Stretching of aromatic C-H | - | - | 3060 | - |
| Stretching of alifatic C-H |  |  | 2919  2832 | 2922 |
| O-H deformation of water | 1647 | - | - | - |
| Stretching of aromatic ring | - | - | 1630  1606  1499  1444 | 1608  1501  1443 |
| N-H scissoring |  |  | 1586 | 1590 |
| Stretching vibration of C-N | - | - | 1250  1211 | 1256  1214 |
| Bending vibration of C-N-C |  |  | 1347 | 1348 |
| Stretching vibration of C-O | - | - | 1097 | 1087 |
| Si-O stretching of outer surface groups | 1117 | 1117 | - | 1118 |
| Si-O-Si stretching of outer surface groups | 1011 | 997 | - | 1010 |
| Al-O-H bending of inner surface groups | 902 | 905 | - | 908 |

**Table S*2*.** Comparison of the time required to deliver 50% of the dose (td 50%) from all samples in pH 1.45, pH 4.5, pH 6.8 and deionized water (NR: 50% Not Reached)

| **Samples** | **td 50% (mean ± SD) in minutes** | | | |
| --- | --- | --- | --- | --- |
|  | **pH 1.45** | **pH 4.5** | **pH 6.8** | **H_2_O** |
| **C** | 2.1 ± 0.9 | 70.1 ± 0.7 | NR | NR |
| **CH** | 5.3 ± 2.9 | 23.4 ± 8.4 | 49.8 ± 7.7 | 135 ± 12.7 |
| **CH_HCl_2M** | 4.8 ± 0.7 | 26.2 ± 1.4 | 48.5 ± 2.1 | 108 ± 2.7 |
| **CH_HCl_4M** | 3.0 ± 1.3 | 12.5 ± 1.6 | 20.2 ± 5.6 | 75.1 ± 3.1 |
| **CH_HCl_6M** | 2.5 ± 0.7 | 7.2 ± 0.4 | 22.3 ± 7.6 | 110 ± 4.2 |
| **CH_HCl_8M** | 3.3 ± 1.3 | 7.5 ± 2.1 | 24.0 ± 4.2 | 81.0 ±4.2 |
| **CH_HCl_12M** | 4.6 ± 2.4 | 14.7 ± 2.3 | 32.0 ±1.1 | 101 ± 1.4 |
| **CH_H_2_SO_4__0.5M** | 2.7 ±0.4 | 11.1 ± 3.2 | 29.4 ± 7.6 | 92.1 ± 5.5 |
| **CH_H_2_SO_4__1M** | 4.7 ± 1.1 | 40.2 ± 1.6 | 300 ± 2.3 | 390 ± 4.3 |
| **CH_NaOH_0.5M** | 6.0 ± 0.8 | 34.2 ± 5.9 | 132 ± 8.5 | NR |
| **CH_PM** | 11.1 ± 0.5 | 22.5 ± 1.5 | 240 ± 2.1 | NR |
| **CH_HCl_8M_PM** | 4.0 ± 0.9 | 16.6 ± 0.9 | 340 ± 1.9 | NR |
| **CH_H_2_SO_4__0.5M_PM** | ND | 15.3 ± 0.8 | 380 ± 2.3 | NR |
